# The Hospitals and the Epidemic

**Published:** 1918-11-02

**Authors:** 


### THE HOSPITALS AND THE EPIDEMIC.

THE epidemic of what is called "Spanish Influenza" engages, and with very good reason, both the press and the public. It has assumed serious proportions, and little sign of abatement can so far be detected. For the hospitals the problem is a very anxious one. Applications for admission are far in advance of the available accommodation, and many refusals are inevitable. The position is complicated by the question of infection, and in some quarters it is urged that patients with influenza ought not to be admitted to the general wards. Indeed, it has been argued that even patients suffering from pneumonia should be excluded. We think it necessary to enter a protest on this point. With the academic discussion of infection we are not concerned. In this respect a case against pneumonia even under ordinary circumstances may be presented. But in the practical sense of the term pneumonia, whether of influenzal or of other origin, offers no reasonable danger to anyone except the sufferer. And in any event such patients are much less threatening to their neighbours in the well-ventilated wards of a hospital than in small houses and stuffy bedrooms. Hence, even if there be a measure of risk, it is, in our judgment, a risk which the general hospitals ought to be ready to accept. Nay, more: the existing state of affairs gives to the hospitals a special opportunity of emphasising their value to the community. The victims are numerous and the doctors at hand are relatively few and much overworked. Neither medical nor nursing help can be obtained in many instances. Hence, so far from excluding or refusing these patients, we would urge that special opportunities should be created for their accommodation. The reception of patients other than those acutely ill should be postponed, and the beds thus set free should be devoted to the existing situation. The time is one not for careful calculation, but for real and practical service in a pressing and urgent emergency.

We wish to make one other point. On several occasions in these columns we have pointed out the serious consequences to the civil community of the invasion of our voluntary hospitals by the War Office. What was justifiable in the early days of the war is without justification now. The War Office has had abundant time in which to organise proper facilities of its own for the care of sick and wounded soldiers. Yet it has continued to utilise the voluntary hospitals and in a corresponding measure to deprive the civilian poor of the opportunities which are theirs by right. Under the influence of the prevailing epidemic this position carries serious consequences, and the managers of the hospitals cannot escape responsibility. They stand as trustees for the rightful beneficiaries, and it is their duty faithfully to administer the obligation imposed on them. The position is all the more serious seeing that a large proportion of the soldiers now occupying beds in the civilian hospitals do not in the least need hospital treatment. Many of them are convalescents, and require merely rest, fresh air, and good food. Everyone, of course, is anxious that these men shall have in full measure whatever is of benefit to them. But it is little short of a scandal that because the War Office has failed to organise proper arrangements elsewhere the authorities should be allowed for their own convenience to utilise hospital accommodation intended for civilians and urgently needed in the present emergency. We have repeatedly protested against this position, and what is happening to-day shows how fully our attitude was warranted.

